# Comparison of Different Staging Systems Applied to a Cohort of Patients With Oral Tongue and Floor of the Mouth Cancer

**DOI:** 10.3389/froh.2021.737329

**Published:** 2021-09-23

**Authors:** Lorenzo Bresciani, Lorenzo Giannini, Alberto Paderno, Fabiola Incandela, Walter Fontanella, Davide Mattavelli, Cesare Piazza

**Affiliations:** ^1^Pediatric Otolaryngology—Head Neck Surgery, Children Hospital ASST Spedali Civili, Brescia, Italy; ^2^Department of Otorhinolaryngology, Maxillofacial and Thyroid Surgery, Fondazione IRCCS, National Cancer Institute of Milan, Milan, Italy; ^3^Unit of Otorhinolaryngology—Head and Neck Surgery, ASST Spedali Civili of Brescia, Brescia, Italy; ^4^Department of Medical and Surgical Specialties, Radiological Sciences and Public Health, University of Brescia, Brescia, Italy

**Keywords:** TNM, oral cavity, staging, depth of invasion (DOI), extra nodal extension

## Abstract

**Purpose:** The present work compares the effects produced by the application of the 7th edition of the tumor node metastasis (TNM) staging system (TNM7), 8th Edition (TNM8) with its two subsequent revisions, and pN-N+ classification on a cohort of patients with oral tongue and floor of the mouth cancer.

**Methods:** A monocentric cohort of 148 patients was retrospectively analyzed. Patients were staged according to the TNM7, TNM8 and revisions, and pN-N+ classification. Stage migration was assessed and overall survival (OS) analyzed with the Kaplan–Meier method. The pT, pN, and stage stratification was evaluated with univariate and multivariate Cox regression and comparing adjacent categories with the log-rank method.

**Results:** pT3-T4a categories showed significant differences in comparison to pT1-T2 for each staging metric employed in both uni- and multivariate analysis. When comparing adjacent pT categories, OS was significantly different only between pT2 and pT3 categories of the TNM8. Disproportionate patient distribution among pN categories was observed in the TNM8, and stratification was scarce. Conversely, in the pN-N+ classification the difference between pN2 and pN3a categories was significant. Only stage IVa reached statistical significance in TNM7, whereas stage III and above were significant in TNM8 and revisions in both uni- and multivariate analysis. However, no significant difference was noted comparing adjacent stages.

**Conclusion:** The TNM8 pT classification differentiated low- from high-risk diseases. Nonetheless, it failed to separate pT1 from pT2 and pT3 from pT4a categories. Conversely, although TNM8 nodal staging was inaccurate, the number of metastatic lymph nodes was more valuable.

## Introduction

The American Joint Cancer Committee (AJCC)–Union for International Cancer Control (UICC) TNM staging system is a universally known tool that is designed to outline the loco-regional and distant extensions of a tumor and assign a prognosis. Furthermore, TNM is a common language that allows comparison between different datasets. To fulfill these goals, the ideal TNM classification should be easily applicable and reproducible but, at the same time, precise enough to properly stratify patients according to their life expectancy, grouping them into classes which should be internally homogeneous and easily distinguishable from one another.

The 8th edition of the AJCC/UICC TNM (TNM8) introduced major changes in oral cavity squamous cell cancer (OCSCC) staging criteria [1, 2]. In pT classification, the parameter “depth of infiltration” (DOI) was introduced as a classifier for pT1, pT2, and pT3 categories due to the growing evidence of its significant correlation with cervical lymph node metastases and loco-regional relapse [1, 3–7]. The AJCC/UICC staging manual also standardized the measurement of DOI, defined as the length of the plumb line drawn from the “horizon” of the basement membrane of the adjacent squamous mucosa to the deepest point of tumor infiltration [2]. This definition eliminates the variability related to the exophytic or ulcerated components of a tumor, even though it does not take into account other factors potentially distorting the DOI measurement such as extra-tumoral (satellite) microscopic neoplastic foci, perineural invasion (PNI), lympho-vascular invasion (LVI), or microscopically involved deep margins (R1) [8]. Of note, after the first release in 2017, two further revisions of TNM8 were proposed. In the first, published on January 1, 2018 (hereafter, TNM8 January 2018), a DOI > 20 mm was introduced for upstaging the disease to the pT4a category. In the second, published on June 25, 2018 (hereafter, TNM8 June 2018), cancers larger than 4 cm and with DOI > 10 mm were moved into the pT4a category.

The first validation studies suggested that the updated staging criteria introduced by the TNM8 (and updates) produced a significant separation between early (pT1-T2) and advanced (pT3-T4) diseases compared with the previous TNM7 [1, 9–12]. Nonetheless, discrimination between pT1 and pT2 categories remained scarce [9–11, 13, 14]. A relevant open issue is a possibility of assuming specific pT classifications according to tumor subsites, in view of the different clinical significance and prognostic meaning that variables such as DOI or masticatory space involvement can have depending on an oral subsite (i.e., tongue, floor of mouth, or buccal mucosa).

Another major novelty in the TNM8 was the introduction of extranodal extension (ENE) in pN classification for every non-virus-related head and neck malignancy due to its profound impact on regional and distant disease control [3, 15, 16]. Moreover, according to the TNM8 updates, the pathologic evidence of at least one contralateral nodal metastasis with ENE allowed upstaging of nodal status to pN3b. In line with previous classifications, the major emphasis was put on lymph nodes laterality and size, while nodes numerosity was still overlooked. However, increasing evidence shows that the absolute count of metastatic lymph nodes strongly predicts cancer mortality [17–19]. Therefore, an alternative nodal classification (called pN-N+ classification), based on lymph nodes numerosity and ENE, was developed and tested, demonstrating good prognostication capability [17, 20, 21].

In light of these observations, further validation studies are required to test the reliability and efficacy of the new classifications proposed. We focused on the tongue and floor of the mouth OCSCC and restaged our monocentric series to assess the potential of both TNM8 (with its January and June 2018 updates) and pN-N+ classifications in stratifying patient prognosis.

## Materials and Methods

Data were retrospectively collected from the medical records of patients treated at the Fondazione IRCCS, National Cancer Institute of Milan, University of Milan, Italy, from January 2010 to December 2016. The study was approved by the Ethical Committee of the Istituto Nazionale dei Tumori di Milano (protocol number 273/20) and was performed in accordance with the ethical standards of the Declaration of Helsinki and its later amendments. Consent for clinical data collection and publication was routinely acquired at the time of surgical intervention. Inclusion criteria were as follows: [1] first diagnosis of OCSCC of the tongue and/or floor of the mouth, [2] upfront surgical treatment with curative intent performed at our Institution, and [3] availability of histological samples for pathologic reassessment. Exclusion criteria were: [1] recurrent cancer or distant metastasis at presentation, [2] first treatment other than surgery, and [3] diagnosis of carcinoma *in situ*.

Of the 549 OCSCC records, 148 met the aforementioned criteria. Clinical and therapeutic data were collected. Histopathological exams were reviewed and pathological features were noted. When DOI values were lacking, histological samples were reanalyzed by a dedicated head and neck pathologist, and DOI was assigned accordingly. All cases were staged according to the TNM7 and subsequently restaged according to either the TNM8 and its two proposed updates (TNM8 January 2018 and TNM8 June 2018). Nodal staging was also reassigned according to the newly proposed pN-N+ classification (Table 1) [20]. Follow-up data were collected through July 30, 2019. All patients received follow-up examinations for at least 30 months or until death.

**Table 1:** Pathologic T- and N-categories criteria according to the TNM8 and its updates (TNM8 January 2018 and TNM8 June 2018), and the pN-N+ classification.

	**TNM8**	**TNM8 January 2018**	**TNM8 June 2018**
T1	Tumor ≤2 cm, ≤5 mm DOI	No change	No change
T2	Tumor ≤2 cm, DOI >5 mm and ≤10 mm or tumor >2 cm but ≤4 cm, DOI ≤10 mm	No change	Tumor ≤2 cm with DOI >5 mm or tumor >2 cm and ≤4 cm with DOI ≤10 mm
T3	Tumor >4 cm or any tumor with DOI >10 mm	Tumor >4 cm or any tumor with DOI >10 mm but ≤20 mm	Tumor >2 cm and ≤4 cm with DOI >10 mm or tumor >4 cm and DOI ≤10 mm
T4a	Tumor invades adjacent structures only (e.g., through cortical bone of the mandible or maxilla, or involves the maxillary sinus or skin of the face)	Tumor invades adjacent structures only (e.g., through cortical bone of the mandible or maxilla, or involves the maxillary sinus or skin of the face) or extensive tumor with bilateral tongue involvement and/or DOI >20 mm	Tumor >4 cm with DOI >10 mm or tumor invades adjacent structures only (e.g., through cortical bone of the mandible or maxilla, or involves the maxillary sinus or skin of the face)
T4b	Tumor invades masticatory space, pterygoid plates, or skull base and/or encases the internal carotid artery	No change	No change
	**TNM8**	**TNM8 January 2018**	**TNM8 June 2018**
N0	No evidence of lymph nodes metastasis		
N1	Metastasis in a single ipsilateral lymph node, 3 cm or smaller in greatest dimension and ENE (–)	No change	
N2a	Metastasis in a single ipsilateral or contralateral node 3 cm or smaller in greatest dimension and ENE (+); or a single ipsilateral node larger than 3 cm but not larger than 6 cm in greatest dimension and ENE (–)	No change	
N2b	Metastasis in multiple ipsilateral nodes, none larger than 6 cm in greatest dimension and ENE (–)	No change	
N2c	Metastasis in bilateral or contralateral lymph nodes, none larger than 6 cm in greatest dimension and ENE (–)	No change	
N3a	Metastasis in a lymph node larger than 6 cm in greatest dimension and ENE (–)	No change	
N3b	Metastasis in a single ipsilateral node larger than 3 cm in greatest dimension and ENE (+); or multiple ipsilateral, contralateral or bilateral nodes of any size with ENE (+)	Metastasis in a single ipsilateral node larger than 3 cm in greatest dimension and ENE (+); or multiple ipsilateral, contralateral or bilateral nodes any with ENE (+); or a single contralateral node of any size and ENE (+)	
	**pN-N+ classification**		
N0	No evidence of lymph nodes metastasis		
N1	1 N+/ENE-	Metastasis in one lymph node but without extra-nodal extension	
N2	1 N+/ENE+ or 2 N+	Metastasis in one lymph node with extra-nodal extension or two positive lymph nodes	
N3a	3–7 N+	Metastasis in three to seven lymph nodes	
N3b	≥8 N+	Metastasis in eight or more lymph nodes	

## Statistical Analysis

A descriptive analysis of the demographics was performed obtaining the distribution of categorical variables, and mean, median, range, and standard deviation (SD) for continuous variables (Table 2). Migration caused by the restaging process was analyzed for pT, pN categories, and stages. Overall survival (OS) was calculated with the Kaplan–Meier method; entry time was the date of diagnosis, whereas event-free patients were censored at the last follow-up examination. Log-rank test was employed for comparison between pairs of adjacent pT, pN categories, and stages. Univariate survival analysis for pT, pN categories, and stages was performed using a Cox proportional hazard model for each classification considered (Table 1). For other categorical or continuous variables, survival analysis was calculated by univariate models based on log-rank test or Cox proportional hazard model, respectively. Variables showing statistical significance on univariate analysis were selected for the multivariate Cox proportional hazard model based on clinical relevance and redundancy. The number of factors employed in multivariate analysis was calculated according to ***Harrell's guidelines***.

**Table 2:** Clinical and pathological data.

Tumor site (%)	
Lateral border of the tongue	84 (56.76)
Oral floor	27 (18.24)
Ventral surface of the tongue	8 (5.41)
Dorsum of the tongue	10 (6.76)
Ventral surface of the tongue and oral floor	19 (12.83)
Age (mean, range, SD)	61.99 (20–92, ±13.97)
Females (%)	59 (39.86)
Males (%)	89 (60.14)
Habits	
Current smoker	66 (44.59)
Former smoker	33 (22.29)
Alcohol drinker	64 (43.24)
Former drinker	9 (6.08)
ECOG performance scale	
Median (range)	0 (0–2)
Histopathology (%)	
SCC	144 (97.3)
Verrucous carcinoma	4 (2.7)
Grading (%)	
Well differentiated (G1)	34 (22.97)
Moderately differentiated (G2)	71 (47.98)
Poorly differentiated (G3)	43 (29.05)
Pathologic features (%)	
PNI	48 (32.43)
LVI	41 (27.70)
Surgical margins (%)	
Negative (R0)	122 (82.43)
Close (Rclose)	8 (5.41)
Microscopically involved (R1)	18 (12.16)
ENE (%)	33 (22.29)
Outcomes (%)	
Non evidence of disease	92 (62.16)
Alive with disease	3 (2.03)
Dead of disease	37 (25)
Dead other causes	14 (9.46)
Lost on follow-up	2 (1.35)

Only patients who underwent neck dissection were considered for univariate comparison between pN categories. Conversely, all patients were analyzed in multivariable analysis, and patients who did not undergo neck dissection because of a low DOI and in whom it did not recur in the neck were grouped together with pN0 cases. In fact, as neck dissection is not mandatory for early OCSCC, the exclusion of the aforementioned cases would lead to a worsening of prognosis for early cancers, influencing the comparison between groups.

Statistical analysis was performed using Stata Statistical Software: Release 15 (StataCorp LLC; College Station, TX). All statistical tests were two-sided, and the level of significance was set at 0.05.

## Results

The analysis included 148 patients, of whom 89 (60.1%) were males, with a mean age of 62 years (range, 20–92; SD ± 13.9). Mean length of follow-up was 51 months (range, 2–113; SD ± 29.1). At the end of the observation period, 95 (64.2%) patients were alive, three (2%) with an evidence of disease. Overall, 51 (34.5%) deaths were recorded, of which 37 (25%) were cancer-related. Two (1.3%) patients were lost to follow-up. Cancer relapsed in 52 (35.1%) patients.

One hundred five patients underwent neck dissection, either therapeutic (75, 50.7%) or elective (30, 20.2%). Neck dissection was not required in 43 (29.1%) patients. None showed nodal relapse within 2 years from diagnosis and were therefore considered pN0 for the purposes of comparison in multivariable analysis.

Microvascular free flap reconstruction was needed in 72 cases (48.6%). Thirty-eight patients (25.7%) received adjuvant radiotherapy (RT), while 26 (17.6%) also received concomitant chemotherapy (CHT). One patient (0.6%) underwent exclusive adjuvant CHT. Further details are shown in Table 2.

**Figure 1:**
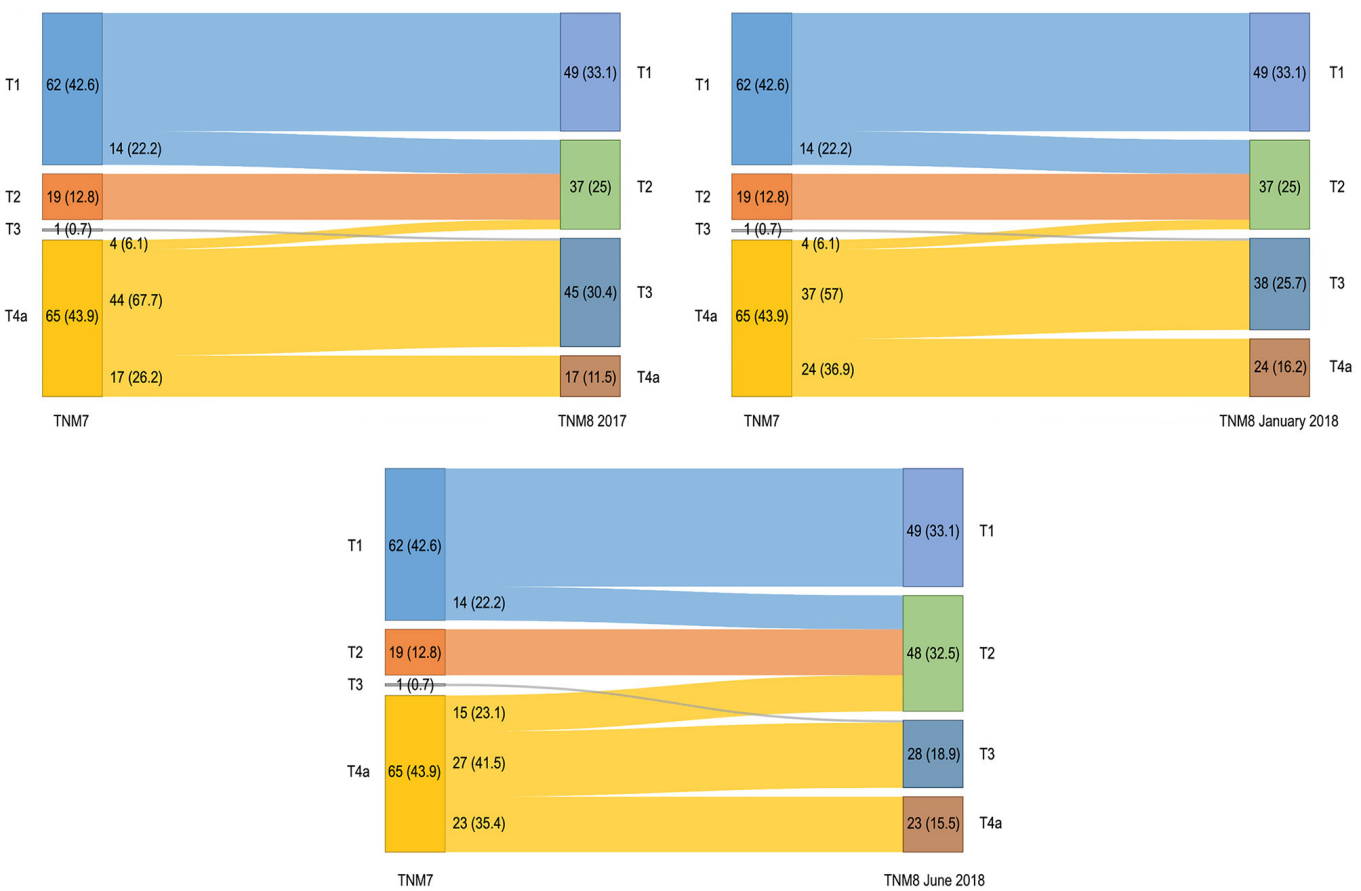
Alluvial diagrams representing migrations and changes in overall pT classification between the TNM7 and all the TNM8 revisions. Numerosity and percentage (between brackets) are reported for each group.

**Figure 2:**
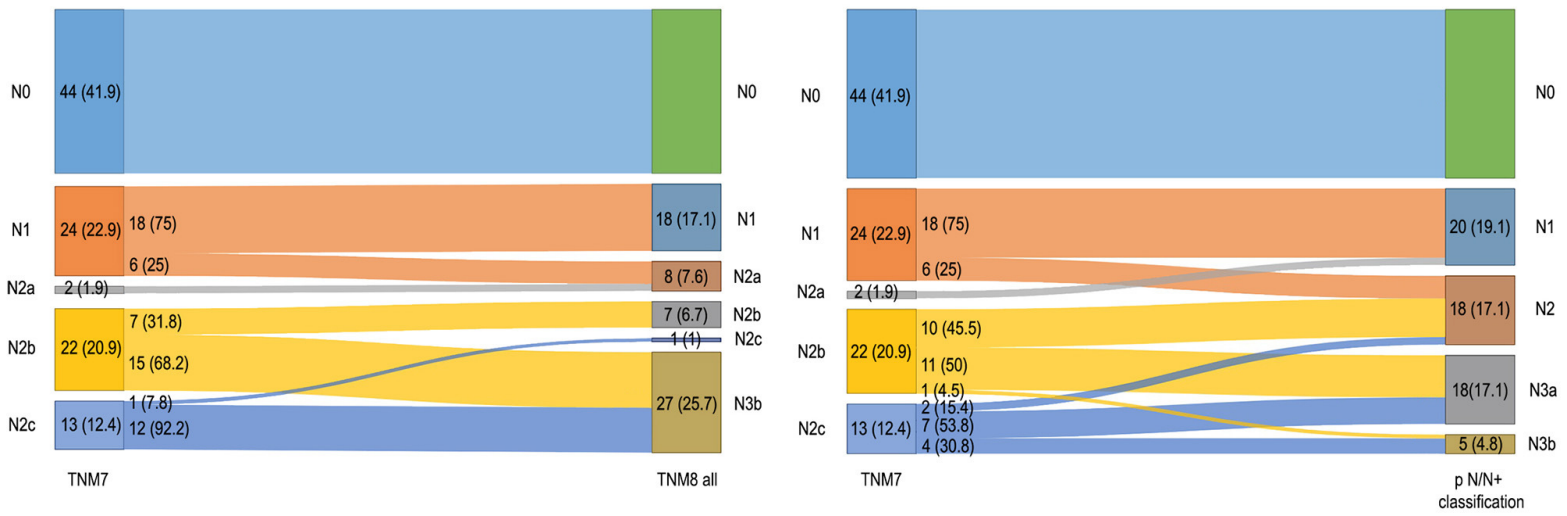
Alluvial diagrams representing migrations and changes in overall pN classification between the TNM7 and TNM8 (left) and between the TNM7 and pN-N+ classification (right). Only patients who underwent neck dissection are taken in consideration (*N* = 105). Numerosity and percentage (between brackets) are reported for each group.

The pT–pN distribution and migration are shown in Figures 1, 2. Primary tumor upstaging occurred in 14 (9.5%) patients (in all cases pT1 moving to pT2). Upstaging remained unchanged through both the TNM8 updates (TNM8 January 2018 and TNM8 June 2018). In contrast, downstaging from pT4a to pT2 and pT3 categories occurred for 48 (32.4%), 41 (27.7%), and 42 (28.4%) patients after restaging by TNM8, TNM8 January 2018, and TNM8 June 2018, respectively (Figure 1). Distribution of cases among pT categories was remarkably more proportionate in TNM8 and updates than in TNM7.

After restaging according to the TNM8 criteria, nodal category was upstaged in 33 (31.4%) patients, due to evidence of ENE. Conversely, no downstaging occurred. Moreover, pN distribution was identical among all the TNM8 revisions (Figure 2). Of note, no pN3a was recorded according to the TNM8. When moving from the TNM7 to the pN-N+ classification, 29 (27.6%) patients were upstaged, whereas two (1.9%) were downstaged (Figure 2).

**Figure 3:**
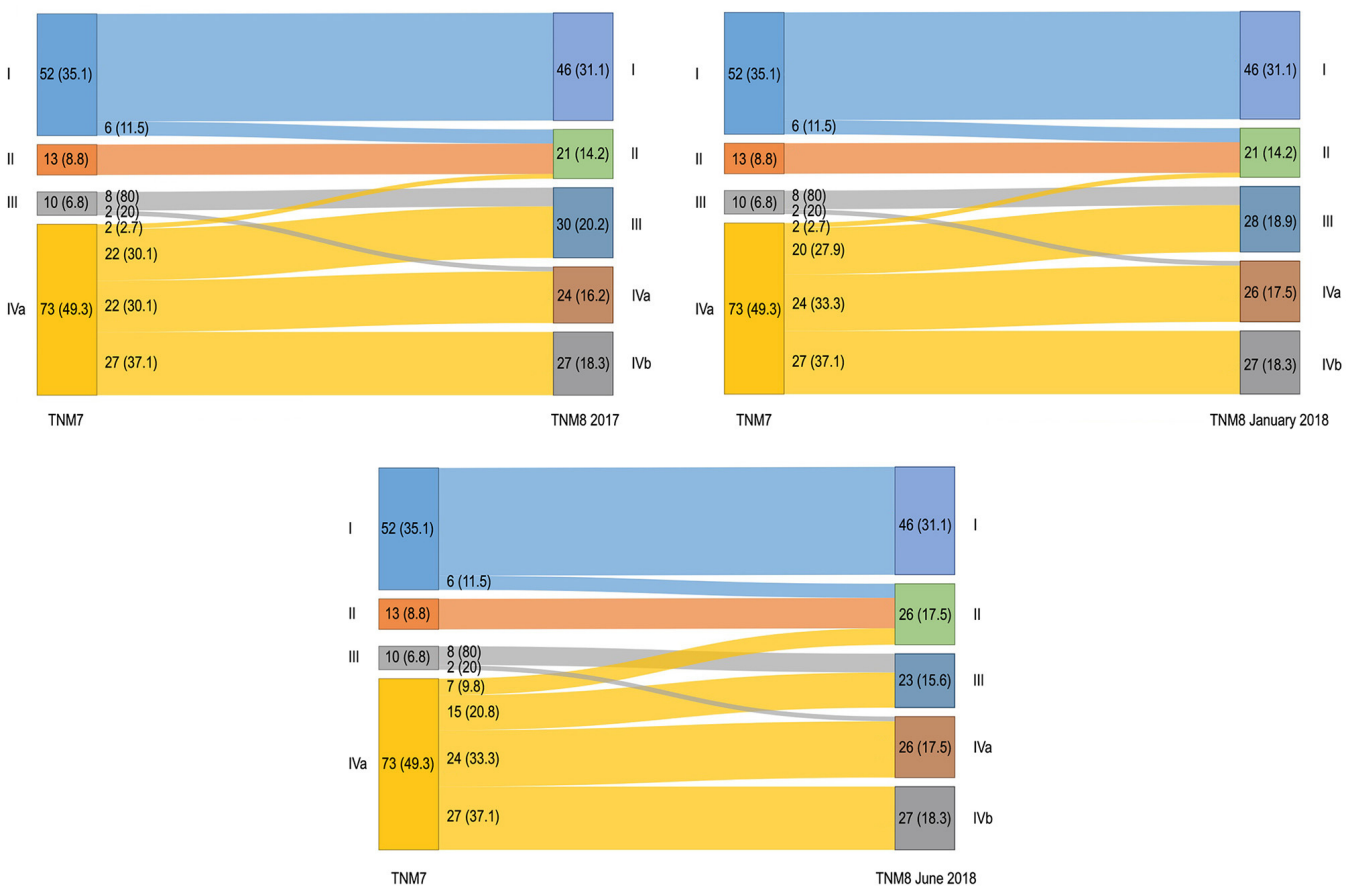
Alluvial diagrams representing migrations and changes in overall stage between the TNM7 and all the TNM8 revisions. Numerosity and percentage (between brackets) are reported for each group.

Stage distribution is shown in Figure 3. No patient was assigned to stage IVb in TNM7. Upstaging occurred for 35 (23.6%) patients and remained unchanged for both TNM8 updates. Downstaging occurred for 24 (16.2%) patients in TNM8 and for 22 (14.9%) patients in both the TNM8 updates. Moreover, patients never changed from early (stages I–II) to advanced (stages III–IV) disease; conversely, two (2.7%) patients moved from advanced to early disease in TNM8 and TNM8 January 2018, and seven (9.8%) did the same in TNM8 June 2018 (in all cases from stage IVa to stage II).

**Figure 4:**
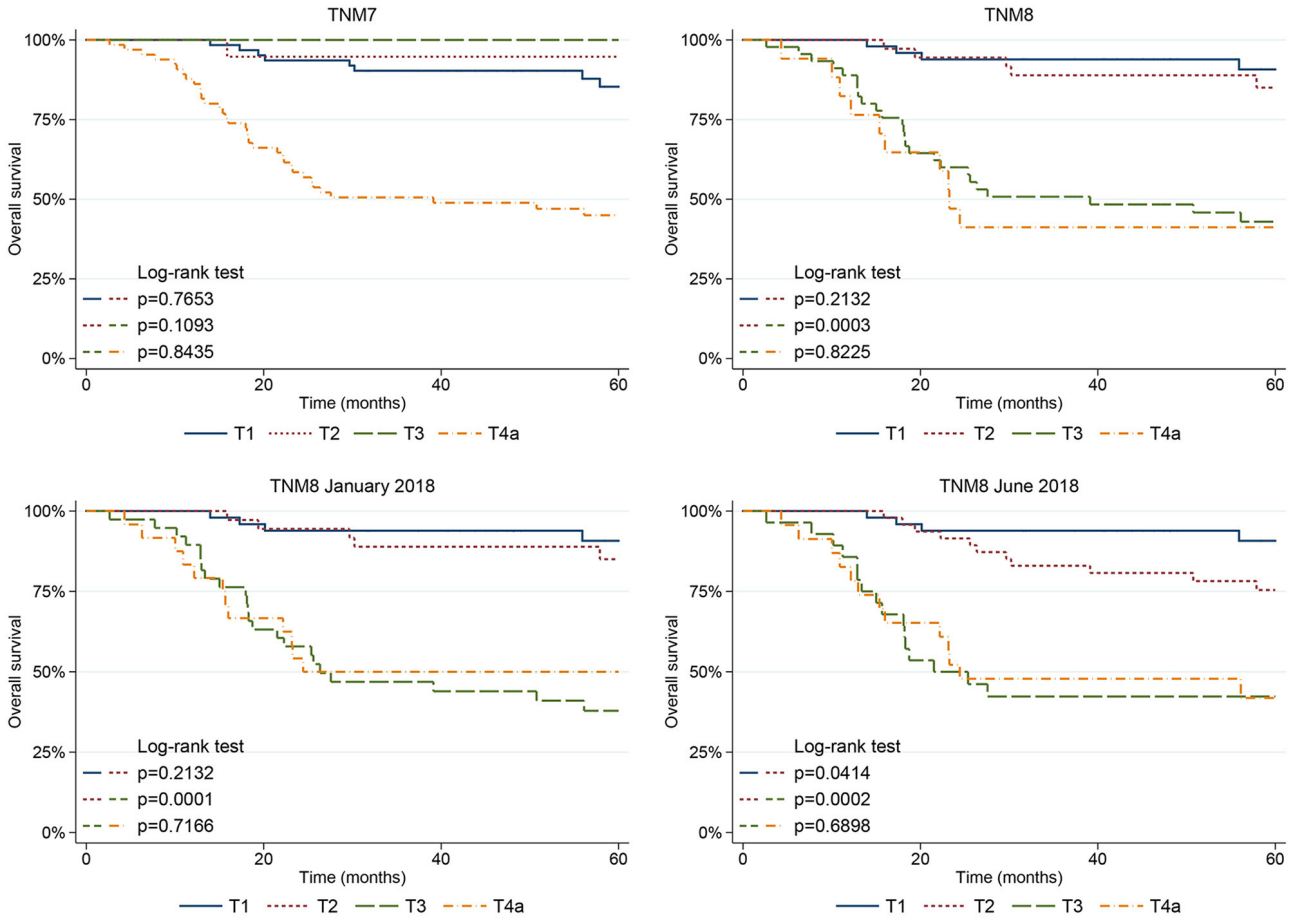
Five-year OS plots stratified for pT category according to the TNM7, TNM8, and subsequent TNM8 updates.

**Figure 5:**
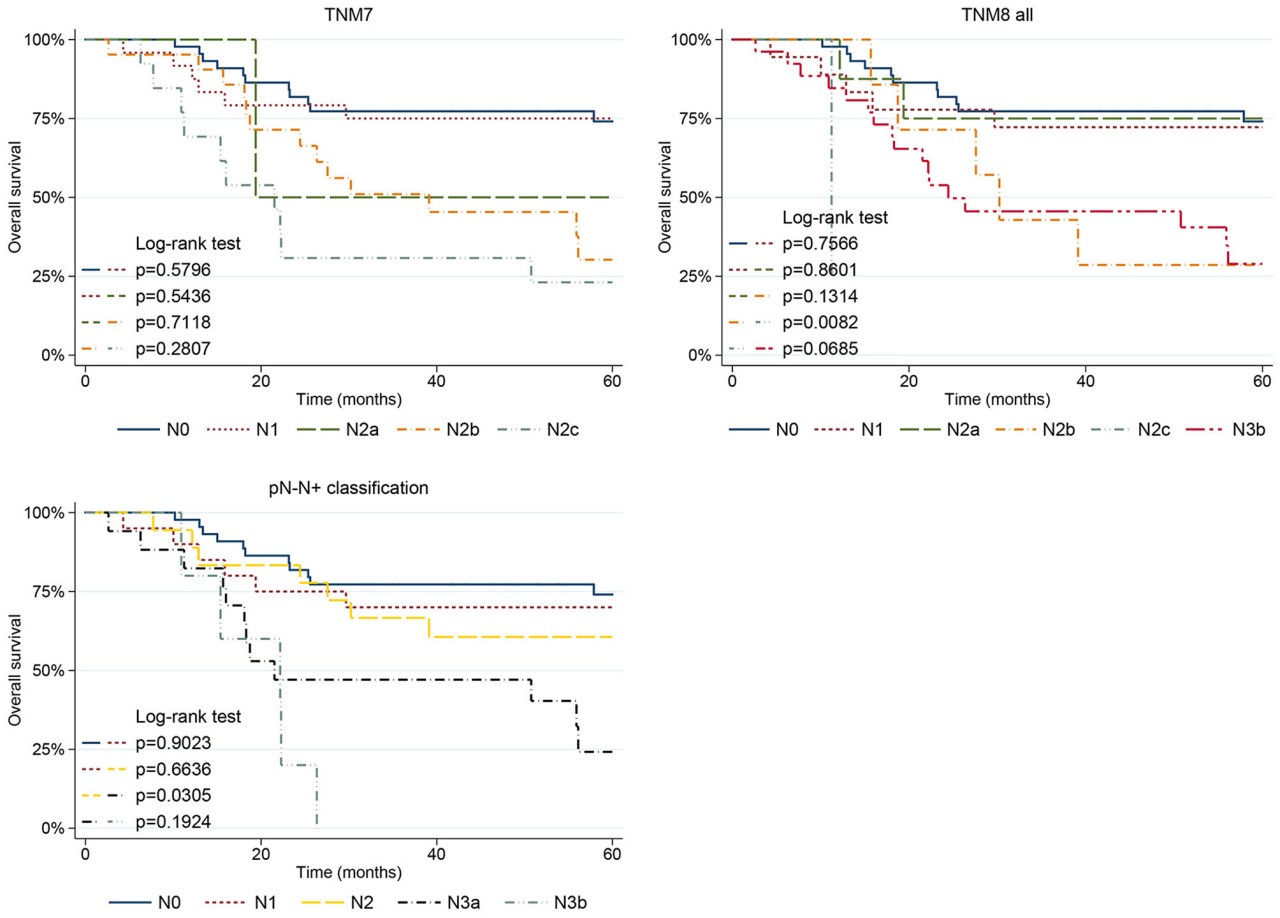
Five-year OS plots stratified for pN category according to the TNM7, TNM8, and pN-N+ classification.

Five-year OS for pT and pN classifications are shown in Figures 4, 5. TNM7 stratification proved scarce, as only the pT4a category showed significantly poorer OS (*p* < 0.001) in univariate analysis, whereas the differences between pT1 vs. pT2, pT2 vs. pT3, and pT3 vs. pT4a categories did not reach statistical significance (Figure 4).

The TNM8 and its updates performed better as pT3-T4a categories always showed statistically significant poorer OS (*p* < 0.001) in univariate analysis. When applying the log-rank test, the pT2 and pT3 categories of the TNM8 and updates showed significantly different OS; conversely, no difference was noted between pT1 and pT2 and between pT3 and pT4a (Figure 4). The TNM8 June 2018 pT2 category also showed significantly poorer OS compared to pT1 (*p* = 0.041) due to the lack of an upper threshold of DOI among the pT2 selection criteria (Figure 4).

When analyzing the pN parameter, in TNM7, the pN2b (*p* = 0.013) and pN2c (*p* = 0.001) categories showed significantly poorer OS. Moreover, no difference in OS was seen between adjacent pairs of pN categories, even though comparison with pN2a was limited as it included only two patients (Figure 5). Moving to the TNM8, the pN2c (*p* = 0.005) and pN3b (*p* = 0.002) categories showed poorer prognosis, whereas pN2b was no longer significant. Only pN2b and pN2c (*p* = 0.008) categories showed significantly different OS, whereas the remaining pairwise comparisons of nodal TNM8 categories were not significant (Figure 5). However, the validity of comparison is limited because pN2a, pN2b, and pN2c categories were scantly represented in TNM8 as many patients moved to pN3b with restaging. Moving to the pN-N+ classification, the N3a (*p* = 0.001) and N3b (*p* < 0.001) categories showed significantly worse OS. However, a significantly different OS between adjacent pN categories emerged only when comparing N2 and N3a categories (*p* = 0.030) (Figure 5).

In univariate analysis, the presence of PNI (*p* < 0.001), LVI (*p* < 0.001), and microscopic positive margins (*p* = 0.003) were associated with poorer OS.

**Figure 6:**
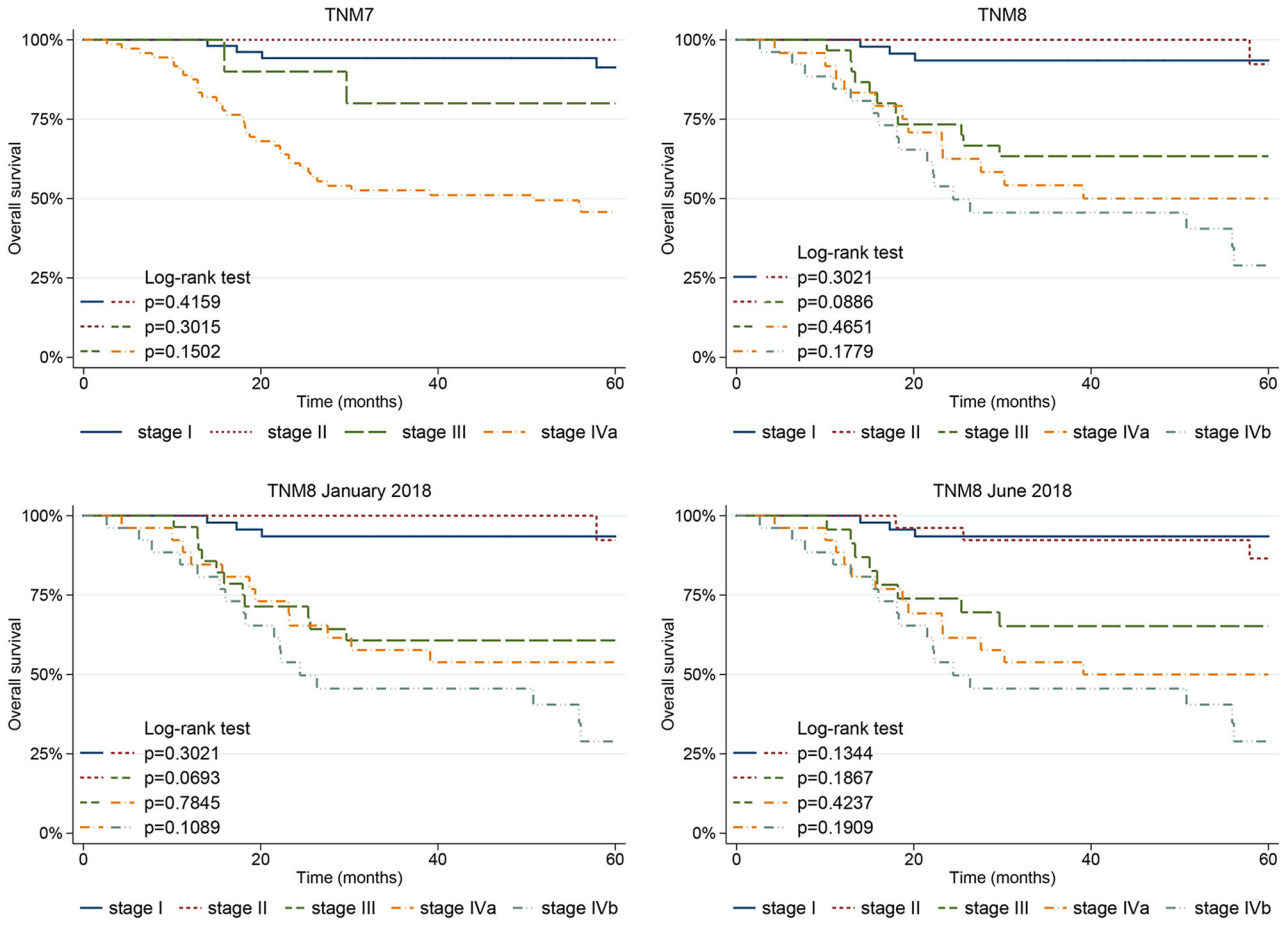
Five-year OS plots stratified according to the stage based on the TNM7, TNM8, and subsequent TNM8 updates.

Five-year OS according to stage is shown in Figure 6. Only stage IVa was associated with poorer prognosis (*p* < 0.001) in TNM7. Conversely, stages III, IVa, and IVb showed poorer OS in TNM8 (*p* = 0.004, *p* = 0.001, and *p* < 0.001, respectively), as well as in TNM8 January 2018 (*p* = 0.003, *p* = 0.001, and *p* < 0.001, respectively), and in TNM8 June 2018 (*p* = 0.008, *p* < 0.001, and *p* < 0.001, respectively). No significant difference in terms of OS was observed between adjacent stages, nor in the TNM7, TNM8, and updates.

In multivariate analysis, pT4a category was associated with lower OS (*p* = 0.032) in TNM7, whereas pT3 failed to reach statistical significance (*p* = 0.051); however, only one patient was classified as pT3, thus limiting the validity of comparison. pT3 and pT4a categories were associated with poorer OS in TNM8 and revisions, and when combining the pN-N+ metrics. The TNM7 pN2b and pN2c categories (*p* = 0.046 and *p* = 0.039, respectively) remained significantly affected by worse prognosis even in multivariate analysis. Conversely, when testing the pN parameter of the TNM8 and updates in multivariate analysis, only TNM8 January 2018 pN3b category significantly predicted a poorer OS (*p* = 0.048). When analyzing the pN-N+ classification, advanced nodal categories, namely N3a and N3b, were statistically significant in multivariate analysis (*p* = 0.022 and *p* = 0.002, respectively).

## Discussion

Our study demonstrated that TNM8 improved OCSCC prognostic stratification and provided better distribution of cases among the different pT categories. In contrast, for what concerned nodal classification, pN-N+ proved to be more balanced (without any empty class) and apparently showed good prognostic stratification, further supporting the opportunity of including a number of positive nodes in future revisions of N classification.

The TNM7 lacked prognostic accuracy when applied to OCSCC, especially for subsites like the mobile tongue and floor of the mouth. The superficial diameter alone was unable to differentiate between early cancers at low-risk of nodal metastases, advanced cancers, and at high-risk of regional disease [13, 22]. Moreover, an inclusion criterion of the pT4a category, namely extrinsic tongue muscle invasion, produced relevant classification bias. In fact, extrinsic muscles run quite superficially at specific sites of the mobile tongue and oral floor (i.e., the genio-glossus at the level of the lingual ventral surface or the palato-glossus and stylo-glossus in correspondence of the amigdalo-glossus sulcus and lateral border of the tongue) so that small and shallow tumors often harbor precocious infiltration of extrinsic tongue muscles [22–24]. Therefore, many tumors ended up being classified as pT4a, although they were relatively small and superficial, whereas very few cases were assigned to the pT3 category, where the purely bidimensional criterion of >4 cm turned most of them into the pT4 group. Furthermore, the distinction between intrinsic and extrinsic muscles was barely impossible for pathologists, and so the classification relevantly relied on the interpretation of the pathological report of the surgeon and preoperative imaging.

The introduction of DOI was meant to provide an objective parameter that could convey the risk of regional spread and local recurrence, and possibly guarantee a better stepwise stratification in prognostic class. Overall, pT category migration was mostly downstaging and never caused the change from early to advanced cancers. Fourteen patients consistently moved from the pT1 to pT2 category. Whereas pT3 category was utterly under-represented in the TNM7, it remarkably grew in TNM8 and revisions, as many cancers showing extrinsic tongue muscles invasion moved from pT4a to pT2 and pT3 categories after restaging. A stronger migration occurred in TNM8, whereas migration was somewhat mitigated by the introduction of adjunctive criteria defining the pT4a category in the following revisions. Other papers reported similar results [9–11, 13, 25–27].

The major effect produced by the introduction of DOI is the clear, nearly dichotomic, prognostic distinction between early (pT1-T2) and advanced (pT3-T4a) diseases, as noted by other authors [9–11, 13]. In fact, the difference in OS between pT2 and pT3 is always significant in TNM8 and both its subsequent revisions (Figure 4). Indeed, in patients affected by OCSCC of the tongue, 10 mm seems to be the DOI cut-off capable of predicting a drop in disease-specific and disease-free survivals [9, 14, 28–30]. These results agree with the anatomic observation that extrinsic tongue muscles are highly represented at a depth >10 mm from the mucosal surface of the tongue and oral floor [20, 22]. Therefore, tumor invasion of these structures could explain the unpredictable local progression of disease through the invasion of muscle fibers and neuro–vascular bundles placed in the lateral and paramedian fibrofatty septa, with ensuing tumor involvement of extra-tongue structures such as the hyoid bone, mandible, and Riolano's group of muscles up to the styloid process [22, 31].

However, TNM8 still failed to depict a homogenous reduction in survival as pT category increases. In fact, the difference in OS between pT1 and pT2 was never statistically significant, except for the TNM8 June 2018. This observation is justified by the increased migration of small (<2 cm) but deeply infiltrating lesions from pT4a to pT2 in TNM8 June 2018, as an upper DOI limit among the pT2 criteria is lacking. The lack of an adequate prognostic stratification for early-stage (pT1 vs. pT2) OCSCC is frequently reported from TNM8 validation studies [9–11, 13, 32, 33]. Apparently, these results are in contradiction with the fact that, in TNM8, patients affected by more infiltrating diseases move from pT1 to pT2, according to their DOI. As DOI correlates with the probability of occult nodal metastases, a cut-off able to separate patients at low- and high-risk of nodal metastases could enhance the prognostic performance of the TNM8 [5–7, 11, 28, 29, 34]. Moreover, in cN0 patients, the risk of regional recurrence within the first 24 months grows as DOI increases, with a steeper curve for values between 2 and 6 mm [5, 6]. However, the DOI employed as cut-off to distinguish patients at low- and high-risk of nodal metastases varies considerably across studies (usually between 3 and 10 mm) [5, 7, 28, 34, 35]. Moreover, the probability of occult nodal metastases in early staged OCSCC varies depending on the subsite, where nodal metastases can be found in 11.2% of OCSCC of the tongue and in 41.7% of floor of the mouth tumors [36]. Finally, in the TNM8, pT1 have a low, but not irrelevant, risk of occult nodal metastases. In fact, the DOI cut-off (5 mm) chosen to separate pT1 from pT2 cancers is higher than that usually employed in clinical practice to decide whether or not to perform prophylactic neck dissection (usually 3–4 mm) [7, 28]. However, Almagush et al. observed that, even on lowering the DOI cut-off of pT2 category to 2 mm, no difference could be observed between pT1 and pT2 [10].

Inclusion criteria of pT4a category were modified in the TNM8, and further revised in its subsequent updates, under the assumption that DOI might influence prognosis even in advanced diseases as, arguably, cancers larger than 4 cm or with a DOI >20 mm actually involve half of the tongue body and floor of the mouth. The real magnitude of these changes is still unknown even though Liao et al. recently observed that all the TNM8 revisions proposed are able to identify high-risk patients (pT4a) [12]. According to our data, pT4a category showed a significantly worse prognosis in both uni- and multivariate analysis. However, no statistically significant difference was noted when comparing the survival of pT3 and pT4a patients, suggesting that TNM8 is still not able to properly stratify the prognosis of locally advanced cancers. This effect might depend on the fact that, in pT3-T4a tumors, the prognostic burden related to the high prevalence of nodal metastases dominates the role played by advanced local extension.

Despite the introduction of ENE, TNM8 still mainly relied on lymph nodes size and laterality, rather than numerosity and, therefore, retained much of the TNM7 intrinsic flaws. The TNM8 pN3a category included nodal metastases larger than 6 cm without ENE which are, indeed, quite uncommon (if not anecdotal) for non-virus-related head and neck SCC, whereas the pN2b category encompassed any number of positive lymph nodes greater than one (without ENE in TNM8). As a consequence, the TNM8 pN2b and pN2c were severely underrepresented (seven and one patients, respectively) because multiple nodes positivity is often associated with ENE, whereas no patient was assigned to TNM7 pN3 and TNM8 pN3a categories. Several other studies, including large validation cohorts of OCSCC patients, have reported this issue [1, 12, 14, 20]. According to our data, the prognostic stratification proved scarce for both TNM7 and TNM8, as the difference between adjacent pN categories was significant only between pN2b and pN2c in TNM8. However, the low numerosity of these categories limited the validity of the comparison.

The pN-N+ classification aimed at overcoming the flaws of the conventional AJCC/UICC TNM nodal staging, neglecting size and laterality, while taking into consideration metastatic lymph nodes count and ENE [17, 20]. In fact, according to Ho et al., while size and side of nodal metastasis lose significance, lymph nodes count still remains a reasonable prognosticator of poor survival in multivariate analysis [20]. As a first result, in our cohort, all the pN-N+ categories were well represented. However, only patients with a high metastatic nodal count, namely those included in the N3a and N3b categories, showed a significantly worse prognosis. This is in line with other reports which demonstrated that the negative prognostic impact of a high number of positive nodes can also outweigh the detrimental effect of ENE [19]. While the TNM pN lacked in prognostic stratification, the pN-N+ classification succeeded in creating a clear distinction at least between patients with low- and high-nodal disease burden, namely between N2 and N3a categories, while the difference between N1 and N2, and between N3a and N3b, was not significant. A threshold of 3 or more nodal metastases, employed for upstaging from N2 to N3a, efficiently predicted a significant decrease in OS even among patients with neck metastases. This corroborates the observation that a metastatic nodal count equal or >3 requires a multimodal adjuvant treatment due to the increased beneficial effect of adding CHT-RT, instead of RT alone, in the postoperative setting [37]. Therefore, the pN-N+ classification, besides being more user-friendly, produced an overall better stratification than both TNM7 and TNM8.

Some limitations of our study should be mentioned. First, the sample size was limited, and this could explain some difference in stage migration with other published reports. Second, we focused only on the oral floor and mobile tongue OCSCC, which may hamper comparisons with other reports. However, the specificity of oral subsites allowed for a thorough analysis of the impact of DOI on pT classification.

## Conclusions

The introduction of DOI among the TNM8 staging criteria for OCSCC led to an improvement in its prognostic stratification, between early (pT1-T2) and advanced (pT3-T4a) diseases. However, discrimination within these low- and high-risk groups (i.e., pT1 vs. pT2 and pT3 vs. pT4) is still poor and requires further improvements.

The pN-N+ classification is a promising tool in terms of ease of use and prognostic capability, which further supports the need to include the number of positive nodes in future pN classifications. Notwithstanding, further studies are needed to reach a satisfactory stratification among patients with a low (≤ 2) count of nodal metastases.

## Data Availability Statement

The raw data supporting the conclusions of this article will be made available by the authors, without undue reservation.

## Ethics Statement

The studies involving human participants were reviewed and approved by the Ethical Committee of the Istituto Nazionale dei Tumori di Milano (protocol number 273/20). Written informed consent for participation was not required for this study in accordance with the national legislation and the institutional requirements.

## Author Contributions

LB, AP, WF, and CP: conceptualization and methodology. LB, WF, and FI: data collection. LB, LG, and AP: statistical analysis. LB: writing—original draft preparation. AP, DM, and CP: review and editing. WF, DM, and CP: supervision. All authors have read and agreed to the published version of the manuscript.

## Conflict of Interest

The authors declare that the research was conducted in the absence of any commercial or financial relationships that could be construed as a potential conflict of interest.

## Publisher's Note

All claims expressed in this article are solely those of the authors and do not necessarily represent those of their affiliated organizations, or those of the publisher, the editors and the reviewers. Any product that may be evaluated in this article, or claim that may be made by its manufacturer, is not guaranteed or endorsed by the publisher.
